# The development of an automated machine learning pipeline for the detection of Alzheimer’s Disease

**DOI:** 10.1038/s41598-022-22979-3

**Published:** 2022-10-28

**Authors:** Nicholas Chedid, Judie Tabbal, Aya Kabbara, Sahar Allouch, Mahmoud Hassan

**Affiliations:** 1 SynapseBio, New York, USA; 2 MINDig, 35000 Rennes, France; 3 Institute of Clinical Neurosciences of Rennes (INCR), Rennes, France; 4 Univ Rennes, Inserm, LTSI-U1099, 35000 Rennes, France; 5 Azm Center for Research in Biotechnology and Its Applications, EDST, Tripoli, Lebanon; 6 School of Science and Engineering, Reykjavik University, Reykjavik, Iceland

**Keywords:** Machine learning, Alzheimer's disease

## Abstract

Although Alzheimer’s disease is the most prevalent form of dementia, there are no treatments capable of slowing disease progression. A lack of reliable disease endpoints and/or biomarkers contributes in part to the absence of effective therapies. Using machine learning to analyze EEG offers a possible solution to overcome many of the limitations of current diagnostic modalities. Here we develop a logistic regression model with an accuracy of 81% that addresses many of the shortcomings of previous works. To our knowledge, no other study has been able to solve the following problems *simultaneously*: (1) a lack of automation and unbiased removal of artifacts, (2) a dependence on a high level of expertise in data pre-processing and ML for non-automated processes, (3) the need for very large sample sizes and accurate EEG source localization using high density systems, (4) and a reliance on black box ML approaches such as deep neural nets with unexplainable feature selection. This study presents a proof-of-concept for an automated and scalable technology that could potentially be used to diagnose AD in clinical settings as an adjunct to conventional neuropsychological testing, thus enhancing efficiency, reproducibility, and practicality of AD diagnosis.

## Introduction

As global population and life expectancy continue to rise, the number of people suffering from neurocognitive disorders or dementia is expected to grow sharply to 74.7 million individuals by 2030^1^. Alzheimer’s disease (AD) is the most prevalent form of dementia among the elderly population accounting for 60–80% of cases^2^. Despite intensive drug discovery efforts, with 121 unique therapies undergoing clinical trials as registered on clinicaltrials.gov in 2020^3^, there unfortunately remain no treatments capable of slowing progression of the disease^4^.

Currently, neuropsychological tests like the Mini-Mental Status Exam (MMSE)^5^ are the most widely used clinical tools for screening for AD as they are easy to perform and are able to evaluate patients’ cognitive function as well as estimate the severity of their cognitive impairment^6^. However, neuropsychological tests are usually time-consuming, require well-cooperating patients and experienced clinicians, and are subject to clinical bias due to language or educational backgrounds of subjects^7–10^. The sensitivity of these tests to subtle changes in cognition is also questionable. Non-invasive neuroimaging techniques, including magnetic resonance imaging (MRI), computed tomography (CT), and positron emission tomography (PET), can also be used to infer pathophysiological mechanisms of AD^11^. However, these tools are expensive and cumbersome, which poses a significant challenge in low-resource environments. In summary, there are no sensitive, specific, reliable, and easily scalable biomarkers or endpoints that currently exist to guide clinical trials and diagnose AD in clinical settings.

In this context, digital biomarkers are highly valuable as they can provide objective and easily scalable and repeatable measurements to support diagnosis and prognosis and to measure therapeutic outcomes^12^. In AD, these digital outcome measures offer a paradigm shift in how treatment outcomes are assessed especially in very early disease states before the detection window of traditional diagnostic methods^12^. In contrast to traditional neuroimaging approaches or questionnaires, digital biomarkers aim to overcome their inability to show meaningful changes when repeated over short time periods as would occur during clinical trials or during early drug response monitoring^12^.

Electroencephalography (EEG) is a promising digital biomarker modality that offers many advantages over the aforementioned modalities in that it is a non-invasive, cost-effective, language-free, culturally fair, mobile, and brain-based screening tool that could uniquely show therapeutic target engagement in the central nervous system (CNS) at a high temporal resolution. EEG is ubiquitous to neurology departments and not restricted to resource-rich, large academic centers as PET and to a lesser extent MRI. Several studies have been conducted to detect brain alterations in AD patients using EEG^13–15^ and to identify early stage abnormalities in neural function^7,16,17^. Recently, many researchers have proposed an international initiative to include the use of EEG biomarkers in the regulatory requirements and guidelines for AD studies^18^.

Although EEG offers promise as a solution addressing many of the aforementioned shortcomings of other diagnostic modalities, EEGs contain extra-cranial artifacts (most commonly eye movements and muscle contractions) that are potentially confounding and that are clinically identified based on qualitative criteria. Failure to accurately account for these EEG artifacts increases the risk for inter-rater variability and sampling bias^19–21^. While machine learning (ML) has been used to predict Alzheimer’s disease from a variety of input data other than EEGs ranging from SPECT^22^ and MRI^22,23^ to cerebrospinal fluid^22^ and genetic variants^24^, in this study we use ML in combination with EEG. To increase rigor in the interpretation of EEG, ML methods have been proposed to automate the process of artifact removal with high accuracy; in this study our team used a well-validated quantitative and automated ML algorithm that has been previously shown to accurately classify artifactual EEG epochs in awake rodent, canine and humans subjects^25^. Moreover, by transforming EEG data from the time domain to the frequency domain, we developed a complete pipeline to automatically generate quantitative features that could be used to identify biomarkers for neurocognitive disorders and develop ML classifiers of mental states in clinical studies^26^.

Prior studies have used ML approaches to categorize AD patients^27–29^, classification algorithms included decision trees^28^, support vector machines^23^, convolutional neural networks^30^, linear discriminant analysis^27^, multiregression linear analysis^29^, and others to classify AD versus healthy subjects^31,32^. However, caveats include long EEG epochs and the use of high-density EEG systems, a challenge for high throughput screening, a lack of clarity around the artifact removal process, and the use of computationally heavy approaches such as graph theory and ‘black box’ ML models that undermine explainability. Therefore, in this pilot feasibility study, we leverage a fully automated EEG assessment process that ensures highly accurate removal of artifacts^25^ and feature extraction, leading to an objective and statistically-guided classification of AD using 5-min resting state EEG acquired via a low-density EEG system (32 channels) from n = 23 healthy controls (HC) and n = 18 subjects clinically diagnosed with AD.

## Materials and methods

### Participants

Twenty-three non-AD subjects with no primary neurological condition referred to as healthy controls or HC (13 males and 10 females, ages 55–81 years, mean age = 65.6 ± 6.8 years) and 20 subjects with AD (8 males and 12 females, mean age = 75.7 ± 7.5 years) participated in this study. Patients were recruited from the memory clinics of Dar al-Ajaza Hospital, Mazloum Hospital, and Vita Nova Polyclinic in Tripoli, Lebanon, whereas healthy subjects were recruited from the local community. The institutional review board at the Lebanese University, Doctoral School of Science and Technology, approved all the experimental protocols and procedures (agreement number CE-EDST-3–2017). All experiments were performed in accordance with the relevant guidelines and regulations outlined in the IRB, and all subjects or their legal guardians gave their informed consent to participate in the study in accordance with all the relevant IRB guidelines and regulations. After screening of medical history, a cognitive screening test and resting EEG recording were performed for each subject. The mini-mental state examination (MMSE) was used as a clinical index to characterize the global neurocognitive performance of participants within five domains: Orientation, registration, attention and calculation, memory, and language^5^. In this study, MMSE scores for HC subjects ranged between 25 and 30 (mean MMSE = 28.1 ± 1.6), and for AD subjects between 3 and 21 (mean MMSE = 11.6 ± 5.1).

### EEG acquisition and preprocessing

EEG signals were recorded using a 32-channel EEG system (Twente Medical Systems International TMSI, Porti system). Electrodes were placed according to conventional ‘10–20’ montage. Signals were originally sampled at 1000 Hz or 1024 Hz, then downsampled to a common frequency of 250 Hz. During the 5-min resting-state EEG recording, all subjects were sitting in a comfortable chair and were asked to stay relaxed, while keeping their eyes closed without falling asleep. Pre-processing of EEG data, feature extraction, statistics, and machine learning were performed using MATLAB (MathWorks) and scikit-learn^33^. Data from 2 of the 20 patients with AD were excluded given poor EEG signal quality. 14 channels were selected for further analysis 'Fp1', 'Fp2', 'F7', 'Fz', 'F8', 'T7', 'C3', 'Cz', 'C4', 'T8', 'P7', 'P8', 'O1', 'O2'; these channels were selected as the most representative samples after downsampling from 32 channels. Channel selection was modeled after prior literature using relatively small channel numbers in a small sample size population^26^. Overall signal quality and impedance levels for each channel were visually inspected; channels considered to be of low or irretrievable quality were excluded from the study. Three EEG channels in total (from a total of 14 channels × 41 subjects = 574 channels) were excluded from further analysis due to exceedingly high impedance resulting in voltage amplitude out of normal range. Specifically, channels Fz and Cz were removed in one AD patient and channel O1 was discarded in one HC subject. The remaining EEG data were downsampled to a common 250 Hz, and then high-pass (3 Hz) and low-pass (35 Hz) filters were applied. EEG data were then segmented into 1 s epochs. Each epoch was assessed for the presence of artifacts using an automated and previously validated analytical pipeline based on support vector-machines^25^, which removed intervals of 1 s artifact bins, provided that each channel contained at least 1.5 min of artifact-free total duration.

### Feature extraction and statistical analyses

Band-wise Power Spectral Density (PSD) were calculated for all channels from artifact-free epochs using periodograms, which were averaged for each channel within each subject, using the two-sided *periodogram.m* function in MATLAB. The periodogram estimate of the PSD of a signal *xL*(*n*) of length *L* where F_s_ is the sampling frequency can be seen in Eq. (1) below:

1 $$ P_{xx} \left( f \right) = \frac{1}{{LF_{s} }}\left| {\mathop \sum \limits_{n = 0}^{L - 1} x_{L} \left( n \right)e^{{ - j2\pi fn/F_{s} }} } \right|^{2} $$

Averaged periodograms were normalized by dividing each frequency bin by the sum of all bins from 3 to 30 Hz. The band-wise PSD is then calculated by taking the average of all bins within each of the following five frequency bands: Delta (1–4 Hz), Theta (5–9 Hz), Alpha (10–13 Hz), and Beta (14–32 Hz). Mean channel band-pass EEG was then obtained by averaging all 14 channels per subject. Paired two-tailed t-test was used to compare band-wise PSDs between both groups with statistical significance of p < 0.05. Moreover, statistical analysis, as described below, was primarily performed to guide the selection of most significant features for ML. Statistical analysis was conducted using Matlab R2021b Update 3 (https://www.mathworks.com/), Python 3.9.12 (https://www.python.org/), and scikit-learn 1.1.1 (https://scikit-learn.org/stable/).

### Feature selection and machine learning

**Figure 1:**
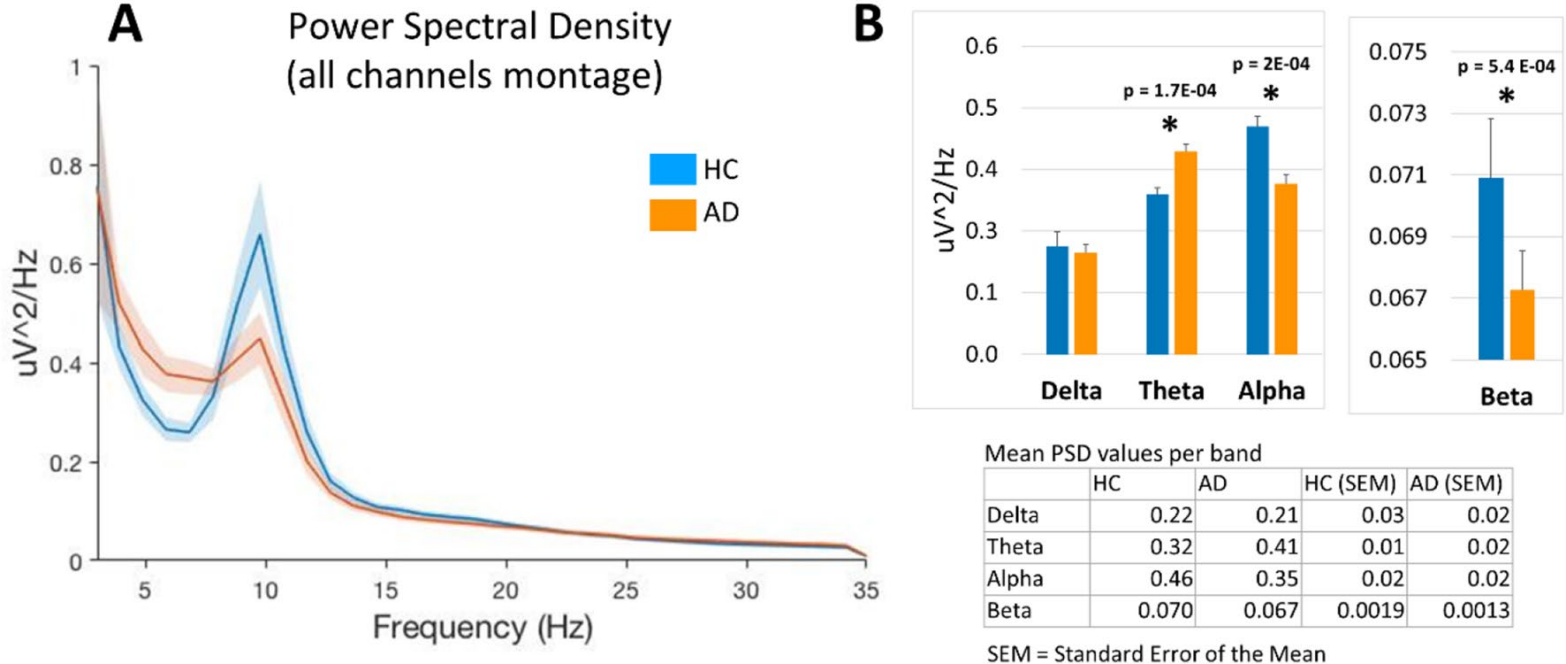
(**A**) Power spectral density (all channel montage obtained by averaging PSD for all channels in each subject) in healthy control subjects (HC, n = 23) and those with Alzheimer’s disease diagnosis (AD, n = 18) in the 3–35 Hz frequency range (shaded areas indicate SEM). (**B**) Mean power in the frequency bands delta (1–4 Hz), theta (5–9 Hz), alpha (10–13 Hz) and beta (14–32 Hz) based on data in panel A (*p < 0.05). Figure generated using Matlab R2021b Update 3 (https://www.mathworks.com/).

**Figure 2:**
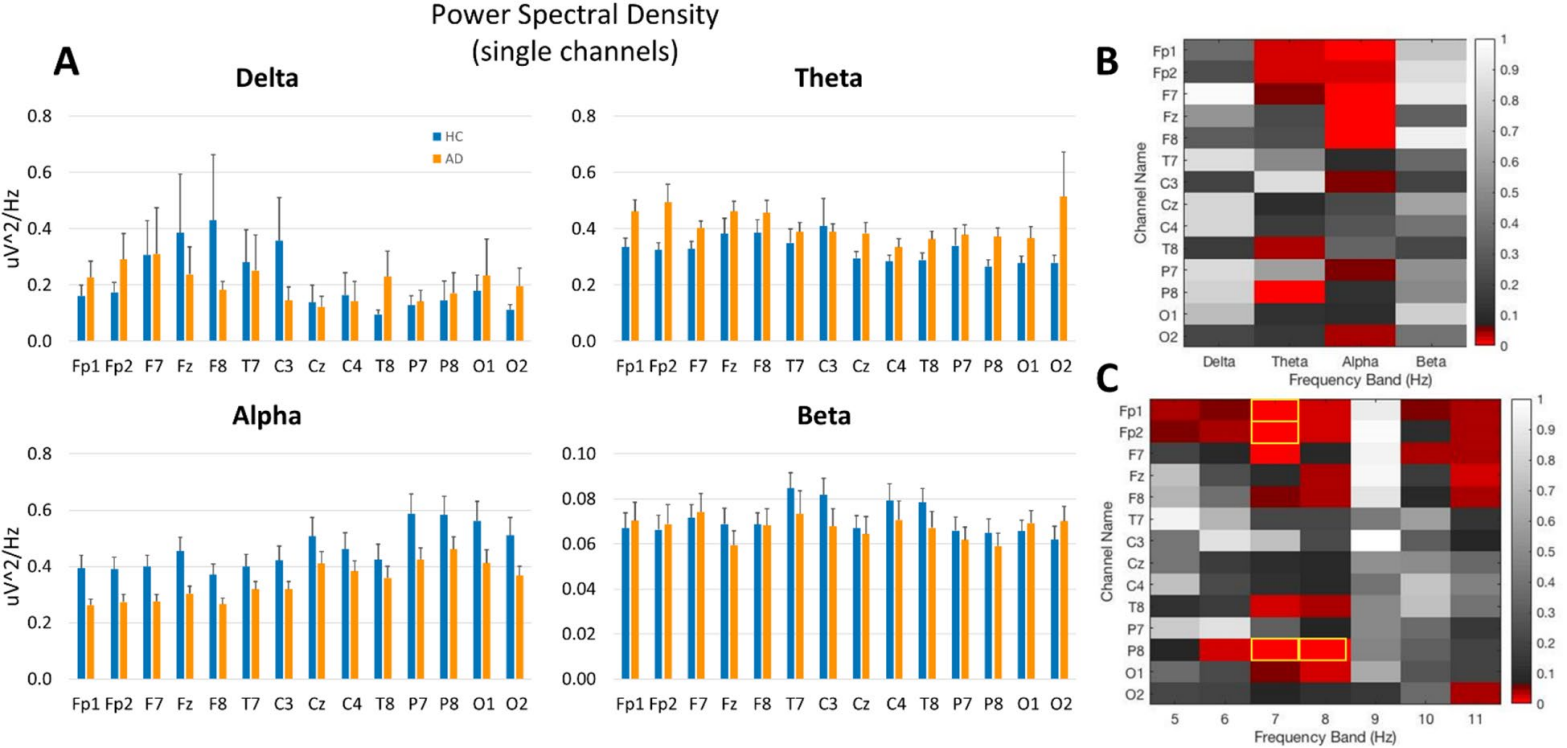
(**A**) Power spectral density in 14 individual EEG channels in HC (n = 23) and AD (n = 18) subjects in the delta, theta, alpha, and beta frequency bands. (**B**,**C**) Heat maps showing t-test values for individual channels in each band (**B**) and for each 1 Hz bin between 5 and 11 Hz (**C**), red hue indicates p < 0.05, and yellow rectangles indicate 4 features selected for machine learning based on the lowest p values. Figure generated using Matlab R2021b Update 3 (https://www.mathworks.com/).

**Figure 3:**
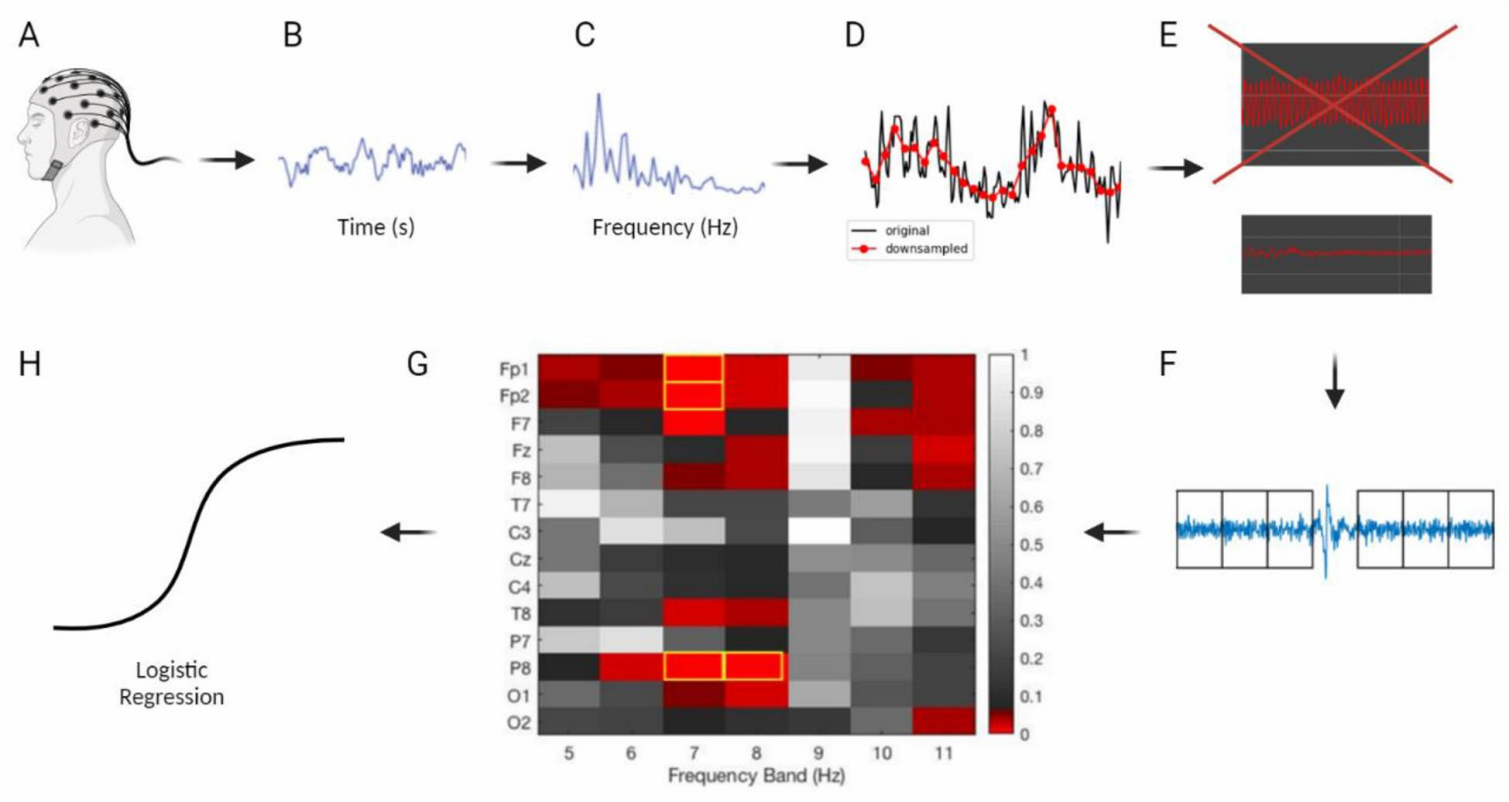
Summary of Automated Pipeline: (**A**) EEG collection (**B**,**C**) transformation from time domain to frequency domain (**D**) frequency downsampling (**E**) removal of low-quality channels (**F**) automated support vector machine-based artifact detection and removal (**G**) feature extraction and selection (**H**) input into ML model (logistic regression). Figure created with BioRender.com.

Only frequency bins between 5 and 11 Hz were considered (see Fig. 1A). Based on PSD plots obtained (see Fig. 2), feature selection was conducted yielding a total of 98 features (7 Hz bins × 14 channels), creating a channel-band feature-set for training binary classification algorithms based on the highest significant difference between both groups. Only the 4 features with the lowest p-values were used (see Results). For machine learning, we used the entire dataset (n = 41) with cross-validation as recent trends in best practices in ML suggest to do^34^. We then explored several classification algorithms on the training data and obtained the best results using Logistic Regression. GridSearchCV was used to test hyperparameters combinations, and classifiers were validated using StratifiedKFold cross validation with k = 5. StratifiedKFold was used to ensure data were properly distributed between folds. Classification accuracy was calculated within the k-folds cross-validation by dividing the number of out-of-sample predicted labels that matched the true label of the sample by the number of total samples (n = 18 AD, n = 23 HC). A summary of these methods can be found in Fig. 3.

## Results

### Power spectral density (PSD) analysis

Quantitative band-wise analysis of the mean PSD in an all-channel montage (obtained by averaging PSD over all channels in each subject) showed significant differences in AD patients as compared to HC (Fig. 1). In particular, in theta band, mean PSD was lower in HC (0.32 ± 0.01) than in AD patients (0.41 ± 0.02), *p* < 0.001, whereas in alpha band, HC showed increased mean PSD (0.46 ± 0.02) as compared to AD patients (0.35 ± 0.02), *p* < 0.001. Similarly, in the beta band, mean PSD in HC (0.070 ± 0.002) was higher than in AD patients (0.067 ± 0.001), *p* < 0.001. Analysis of PSD in individual channels further showed significant changes localized to particular electrodes in distinct bands (Fig. 2 A, B) and frequency bins (Fig. 2C). Based on statistical results, the following 4 PSD features were selected based on the highest significance level (i.e., lowest *p* values): channel P8 at 8 Hz (*p* = 0.001), channel P8 at 7 Hz (*p* = 0.002), channel Fp2 (*p* = 0.003) at 7 Hz, and channel Fp1 at 7 Hz (*p* = 0.009).

### Logistic regression analysis

Using logistic regression, we reached a mean accuracy of 81.11% on the entire dataset (AUC: 86.58%, precision: 78.33%, recall: 75%). Hyperparameters for these results included: C = 1, class_weight = None, fit_intercept = True,max_iter = 400, multi_class = 'auto',penalty = 'l2', solver = 'liblinear'.

## Discussion

In this proof-of-concept study, we demonstrate that the quantitative analysis of brief (5 min), resting-state EEGs in the frequency domain using a portable, low density (14 channels) montage reveals significant differences between AD patients and HC. Moreover, a transparent, explainable machine learning approach, guided by conventional statistical methods to identify relevant data features in specific channels and frequency bins based on empirically significant values, results in classifier models that can distinguish subjects in either HC or AD category with high accuracy.

Alzheimer’s disease is the most common cause of dementia among elderly people but lacks treatments capable of slowing disease progression^1^. The lack of reliable disease endpoints and/or biomarkers contributes in part to the lack of effective therapies^12^. Functional imaging studies might provide insight, however, complementary assessment of brain activity at the speed of neural activity is required. Moreover, a qualitative, definitive, more comprehensive diagnosis of AD, especially at an early stage prior to neural cell death, would open up more possibilities for targeted therapeutic interventions focused on neuroprotection, thus potentially delaying AD progression before major impairments emerge^7^.

Several EEG studies have been conducted to detect abnormalities in brain function in AD patients^13,15,35^, in particular during early stages^7,16,17^. Whereas most studies have focused on the analysis of evoked-related potentials (ERP) in EEG while subjects are engaged in various cognitive tasks to identify the perturbations of specific cognitive processes^16^, experimental constraints imposed by these paradigms might be beyond the tolerance and capacity of elderly subjects. Conversely, resting-state protocols (as in this study) are simpler, shorter and easier to implement. According to various reviews^7,16,18^, the most commonly reported resting-state EEG findings is generalized slowing of brain activity in the frequency domain in AD patients. Specifically, progression to AD is characterized by an increase in low-frequency power (delta and theta bands), accompanied by a decrease in higher frequency power (alpha, beta, and gamma). Our results are largely consistent with these changes; while no significant change was noted between HC and AD patients in the delta bands, we did observe the expected increase in theta bands and a decrease in alpha and beta bands in AD patients.

Importantly, most AD research involving EEG relies heavily on qualitative examination of the raw traces to remove artifacts as a first step, a subjective procedure that undermines rigor. Therefore, an automated, quantitative analysis of EEG is critical for objectivity and reproducibility in the assessment of EEG data. Using an analytical pipeline developed by our team based on ML, our first step in the analysis of EEG is quantitative, automated, and efficient. Moreover, the development of ML techniques has allowed more sophisticated analysis of EEG in the frequency domains, thus allowing for classification methods, such as decision trees, support vector machine, K-nearest neighbors, and linear discriminant analysis to more accurately identify AD patients^27–29^ and distinguish between AD and healthy subjects^30–32^., with some classifiers presumably achieving sensitivity and specificity as high as 90%^35,36^.

Nevertheless, several problems hinder the clinical use of resting-state EEG for AD screening as outlined in our abstract. In this study, we present a fully automated framework that overcomes these issues simultaneously for the first time:

1. a lack of automation and unbiased removal of artifacts—overcome via implementation of automatic artifact removal via SVMs.
2. a dependence on a high level of expertise in data pre-processing and ML for non-automated processes—our analytical pipeline negates the need for such a high level of expertise as described in Fig. 3.
3. the need for very large sample sizes and accurate EEG source localization using high density systems—we demonstrated good results with a sample size of 41 patients using only 14 channels.
4. and a reliance on black box ML approaches such as deep neural nets with unexplainable feature selection—we used PSDs guided by statistics for feature selection for increased interpretability, which was input into a logistic regression model which offers greater interpretability than many more complex ML models.

Despite the promising results, this study still has several limitations. Our sample size was relatively small; large-scale, multicenter data is needed to further assess the generalizability of our model. Second, incorporating multi-modal data into ML models maximizes the chances of discovering meaningful biomarkers^37^. Including vital signs, genetic data, and co-morbidities, along with EEG, may lead to more accurate biomarkers. Third, although the mean age of our study groups was different, the two populations overlapped in age (healthy controls = 65.5 6 ± 6.8 years and AD = 75.7 ± 7.5 years); additionally, this small difference in age is unlikely to be sufficient to explain the significant differences in these patients' EEGs. Finally, our pipeline was not validated on an external, independent dataset, doing so would increase the generalizability of our findings.

Although our machine learning approach could be conceived of as conventional, the novelty in our approach is twofold: (1) “transparent” machine learning techniques as opposed to black box deep learning methods, and (2) preprocessing EEG signals in an automated manner to remove artifacts such that our results are reproducible, rigorous, and scalable. These two novel aspects allowed us to obtain proof of concept data in a relatively small sample size.

In summary, we explored the development of a fully automated discrimination process for AD based on brief epochs of resting-state EEG using low density channel montage, an end-to-end automated analysis pipeline for data preprocessing, and statistically guided feature extraction, leading to explainable ML classification with high accuracy. Therefore, this study presents a proof-of-concept for a scalable technology that could potentially be used to diagnose AD in clinical settings as an adjunct to conventional neuropsychological testing, thus enhancing efficiency, reproducibility, and practicality of AD diagnosis. Further evaluation and testing in larger data sets is required to further validate our results.

## Data Availability

The data comes from memory clinics of Dar al-Ajaza Hospital, Mazloum Hospital, and Vita Nova Polyclinic in Tripoli, Lebanon, whereas healthy subjects were recruited from the local community, and it is not publicly available as it contains sensitive information of patients. Please contact the corresponding author with any further queries regarding data availability.
